# Adipose-Derived Stromal Vascular Fraction/Xenohybrid Bone Scaffold: An Alternative Source for Bone Regeneration

**DOI:** 10.1155/2018/4126379

**Published:** 2018-04-29

**Authors:** Ilaria Roato, Dimas Carolina Belisario, Mara Compagno, Laura Verderio, Anna Sighinolfi, Federico Mussano, Tullio Genova, Francesca Veneziano, Gianni Pertici, Giuseppe Perale, Riccardo Ferracini

**Affiliations:** ^1^Center for Research and Medical Studies, A.O.U. Città della Salute e della Scienza, Turin, Italy; ^2^Department of Chemistry, Materials and Chemical Engineering “Giulio Natta”, Politecnico of Milan, Milan, Italy; ^3^CIR Dental School, Department of Surgical Sciences, University of Turin, Turin, Italy; ^4^Department of Life Sciences & Systems Biology, University of Turin, Turin, Italy; ^5^Pathology Unit, A.O.U. Città della Salute e della Scienza, Turin, Italy; ^6^Industrie Biomediche Insubri SA, Mezzovico-Vira, Switzerland; ^7^University of Applied Sciences and Arts of Southern Switzerland (SUPSI), Manno, Switzerland; ^8^Department of Surgical Sciences (DISC), Orthopaedic Clinic, IRCCS A.O.U. San Martino, Genoa, Italy

## Abstract

Adipose tissue-derived stem cells (ASCs) are a promising tool for the treatment of bone diseases or skeletal lesions, thanks to their ability to potentially repair damaged tissue. One of the major limitations of ASCs is represented by the necessity to be isolated and expanded through *in vitro* culture; thus, a strong interest was generated by the adipose stromal vascular fraction (SVF), the noncultured fraction of ASCs. SVF is a heterogeneous cell population, directly obtained after collagenase treatment of adipose tissue. In order to investigate and compare the bone-regenerative potential of SVF and ASCs, they were plated on SmartBone®, a xenohybrid bone scaffold, already used in clinical practice with successful results. We showed that SVF plated on SmartBone, in the presence of osteogenic factors, had better osteoinductive capabilities than ASCs, in terms of differentiation into bone cells, mineralization, and secretion of soluble factors stimulating osteoblasts. Indeed, we observed an increasing area of new tissue over time, with and without OM. These data strongly support an innovative idea for the use of adipose SVF and bone scaffolds to promote tissue regeneration and repair, also thanks to an easier cell management preparation that allows a potentially larger use in clinical applications.

## 1. Introduction

Regenerative medicine based on stem cell ability to potentially repair injured tissues is a promising treatment for many orthopaedic problems [1, 2]. Indeed, the availability of adult stem cells, such as mesenchymal stem cells (MSCs), which can be easily retrieved by adipose tissue, has dramatically enlarged their potential field of application [3–7]. One of the major limitations of MSCs is represented by the necessity to expand them through *in vitro* culturing, transforming them into a pharmaceutical product with its restrictive regulatory clearance and connected difficulties for clinical routinary use. Thus, a strong interest was generated by the stromal vascular fraction (SVF), the noncultured fraction of MSCs, directly obtained after collagenase treatment of adipose tissue [2]. SVF contains MSCs called adipose tissue-derived stem cells (ASCs), which are able to differentiate in bone, cartilage, and adipose tissue [7, 8] and have been successfully used in human patients without the need of a surgical procedure [9]. In the last decade, many clinical trials tested infusion of ASCs or SVF alone or in combination with platelet-rich plasma (PRP): they not only showed encouraging results in regenerating cartilage in patients with large cartilage lesions or with osteoarthritis (OA) but also report improvement in orthopaedic scores for pain, function, range of motion, and MRI evidence of cartilage regeneration [9–11]. Often in OA, there is a concomitant subchondral bone damage; thus, a role of SVF in regeneration of bone is envisioned. Moreover, other pathological conditions (e.g., osteonecrosis of femoral head, bone fracture, and nonunion fractures) could benefit from the SVF ability to regenerate bone.

In order to improve bone regeneration, different scaffolds have been generated, using different biomaterials, and recent trends point towards a composite approach for best mimicking the human bone structure [12]. In this framework, SmartBone (SB), a xenohybrid bone graft [13], resulted to be particularly efficient: it is commercially available as a medical device, and it was initially developed as a bone substitute for reconstructive surgeries in the presence of bone losses, giving excellent results [13, 14]. SB is constituted of a bovine bone matrix reinforced by a micrometric thin poly(l-lactic-co-*ε*-caprolactone) film embedding RGD-containing collagen fragments (extracted by purified bovine gelatin), which overall results in increased mechanical properties, hydrophilicity, cell adhesion, and osteogenicity [14]. In order to deeply investigate the basic biological mechanisms beneath the recorded clinical performances of such a graft and to investigate the bone-regenerative potential of ASCs and SVF, we studied their ability to colonize SB and generate new tissue when cultured on it [15].

## 2. Materials and Method

### 2.1. Isolation of SVF from Adipose Tissue

SVFs were isolated from fresh adipose tissue derived from 7 patients, who provided written consent according to the approval of the Ethical Committee of our institution. Seven lipoaspirates were processed according to a previously published procedure [3]. Briefly, after enzymatic digestion with Collagenase NB4 (SERVA Electrophoresis) and subsequent washes with saline solution, the cell pellets were treated with a cell lysis solution (Promega) to discard blood cells, then cells were collected and counted. The phenotype of ASCs contained in the SVF was evaluated by flow cytometry, soon after SVF isolation.

### 2.2. Flow Cytometry Analysis

Mesenchymal cell surface markers were analysed by flow cytometry on fresh SVF and on cultured ASCs. A standard labelling protocol was performed with the following antibody fluorochrome-conjugated and isotypic controls: human CD105 PE (Invitrogen), CD73 FITC (kindly provided by Prof. Malavasi, University of Turin), CD44 FITC, CD45 PerCP, CD271 APC, IgG1 PE, IgG1 APC and IgG2a PerCP (Miltenyi Biotec), and IgG1 FITC conjugate (IMMUNOSTEP). About 10^5^ events/sample were used for capture with CellQuest software. All data were analysed with Flowlogic software (Miltenyi Biotec).

### 2.3. Scaffold Preparation

The xenohybrid bone scaffold, SB, was produced according to a previously published method [14]. SB discs (7 × 3 mm—made to fit into multiwells) were washed twice with saline buffer solution then kept in *α*-MEM to improve hydrophilicity and the subsequent cell seeding.

### 2.4. Cell Cultures

To obtain a population of ASCs, the SVF cells were seeded in T25 flasks and cultured in DMEM with 10% FBS, 2 mM glutamine, and 1% antibiotics (Gibco, Life Technologies), and the medium was replaced to eliminate nonadherent cells after 24 h. Cell cultures were maintained, and ASCs at the 2nd passage were utilized for all experimental settings.

SVF and ASCs were cultured on tissue culture plastic and on SB discs at a concentration of 1 × 10^6^ SVF cells and 1 × 10^5^ ASCs for 60 days, in *α*-MEM with 10% FBS, 2 mM glutamine, and 1% antibiotics (Gibco, Life technologies) or in osteogenic medium (OM) containing *α*-MEM supplemented with 10% FBS, 50 *μ*g/ml ascorbic acid, 10^−8^ M dexamethasone, and 10 mM beta-glycerophosphate (Sigma-Aldrich). The medium was replaced twice a week.

At 15, 30, and 60 days, cells cultured on tissue culture plastic were stained for alkaline phosphatase (ALP) according to the kit produced by Sigma-Aldrich to monitor the osteoblast (OB) differentiation. The mineralization activity of OBs was evaluated through the detection of mineralized nodules by von Kossa staining.

### 2.5. Real-Time qRT-PCR

After 15, 30, and 60 days, we isolated cells from SB by treating them with collagenase I (SERVA) for 30 minutes, then cells were washed and dissolved in TRIzol reagent for RNA extraction by the RiboPure kit procedure (Ambion). One microgram of RNA was converted up to single-stranded cDNA by the High-Capacity cDNA Reverse Transcription Kit (Applied Biosystems). Quantitative real-time PCR was performed by the CFX96 system (Bio-Rad). The mRNA expression of the following genes was tested: osteocalcin (OCN, NM_199173.5), alkaline phosphatase (ALP, NM_000478.5), RUNX2 (NM_001024630.3), and collagen 1 (COLL-1, NM_000088.3); the primer sequences of the first 3 genes were previously published [16], whereas we designed and tested COLL-1 FW primer CTGTTCTGTTCCTTGTGTAAC and COLL-1 REV primer GCCCCGGTGACACATCAA. RT-PCR was performed with SensiFAST™ SYBR Hi-ROX kit (Bioline). The amplification protocol foresees 40 cycles with a Tm of 58°C. The expression of *β*-actin was chosen to normalize gene expression data and the 2^−ΔΔCt^ method for the quantitative analysis with CFX Manager software (Bio-Rad).

### 2.6. Micro-CT

SB was analysed by high-resolution X-ray microtomography (SkyScan 1172, Bruker) to study the structure and to compare the volumes of SB before and after SVF and ASC colonization. Acquisitions were performed at 80 kV using a 0.5 mm Al filter at a resolution of 6 *μ*m, 0.4° of rotation step, 360° scan, and 4x frame averaging. Datasets were reconstructed with NRecon software (Bruker), and quantification was performed by two expert operators on axial slices, measuring the mineralized tissue length by using DataViewer software (Bruker). A color contrast mask was used to allow a clear identification of newly formed mineralized tissue.

### 2.7. Scanning Electron Microscopy (SEM) Analysis

As previously shown [14], visual assessment of cell layering on SB was performed via environmental SEM, both on unseeded and seeded samples at 10 kV with EVO 50 EP Instrumentation (Zeiss, Jena, Germany). At the end of the cell culture studies, scaffolds were fixed with 2.5% (*v*/*v*) glutaraldehyde solution in 0.1 M sodium cacodylate buffer for 1 h at 4°C, washed with sodium cacodylate buffer, and then dehydrated at room temperature in a gradient ethanol series up to 100%. At 15, 30, and 60 days, each sample was analysed on the side external surfaces and then halved with a sharp scalpel and the two inner exposed surfaces were internally analysed.

### 2.8. Histochemical Analyses

SB discs were fixed in a neutral buffer containing 4% formaldehyde, washed, and decalcified with MicroDec EDTA-based from Diapath. Specimens were then dehydrated and paraffin-embedded through EZ Prep Concentrate solution (Ventana Medical Systems Inc.). Sections were stained for H&E for morphological analyses. Immunohistochemical analysis (IHC) was performed by the automated instrument BenchMark ULTRA (Ventana). Tissue sections were incubated with the following primary mouse monoclonal antibodies (MoAb) from Abcam: COLL-1 (ab34710, at 1 : 400 dilution), OCN (ab93876, at dilution 1 : 250), and TGF*β* (ab92486, at dilution 1 : 150). They were titrated to yield maximal specific staining and minimal nonspecific or background staining. The endogenous peroxidase activity was inhibited by the addition of ultraView Universal DAB Detection Kit (Ventana). All samples were counter-stained with Mayer's hematoxylin solution (Roche) and mounted with Kaiser's glycerol gelatin. Slides were examined double blind, and microphotographs were taken using an Olympus BX51 microscope equipped with a digital camera (Nikon DCS E995).

### 2.9. ELISA

The expression levels of vascular endothelial growth factor (VEGF) and endothelin-1 (ET-1) in cell culture supernatants were determined by a commercially available Quantikine ELISA kit according to the manufacturer's instructions (R&D Systems). Supernatant samples were collected at days 4, 15, 30, and 60 of culture. Day 4 is the starting point of the analysis and is therefore referred to as day 0 in the graphs: indeed, the cells need to grow for a few days (from day 0 to day 4) and are used for analysis. Samples were assayed in duplicate, and data were expressed as mean values.

### 2.10. Statistical Analysis

All statistical analysis was carried out using GraphPad Prism 4. Data were presented as mean with standard error. Data were analysed by one-way ANOVA with Bonferroni's multiple comparison test. To evaluate significant differences in the means of newly mineralized tissue length, at least 150 bone-like segments for each conditions were measured. Results were considered significant with *p* < 0.05.

## 3. Results

### 3.1. Phenotypical Analysis of SVF and ASCs

**Figure 1:**
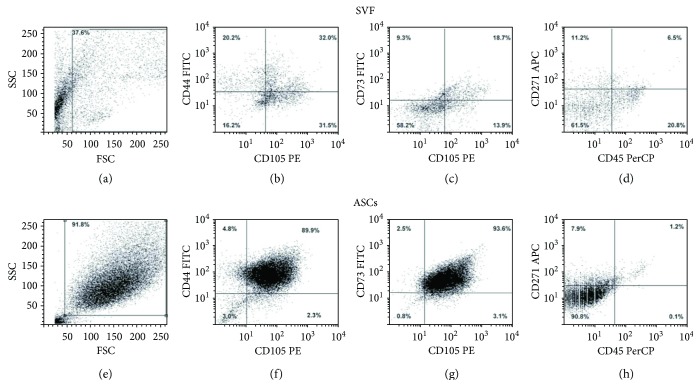
Flow cytometry analysis of mesenchymal cells in freshly isolated and cultured SVF. Dot plots show the morphology of SVF (a) and ASCs (e). SVF is a heterogeneous cell population, containing CD105-, CD44-, CD73-, and CD271-positive mesenchymal cells (b, c) and a small fraction of CD45-positive cells (d), due to the normal presence of leukocytes in SVF. After 15 days of culture, a large and enriched population of ASCs highly expresses mesenchymal markers (f, g), whereas it is completely negative for CD45.

A mean of 7% of ASCs coexpressed CD105, CD44, CD73, and CD271 and were CD45-negative, with a large range of variability (1.6–13.6%) due to human differences. Cells expressing mesenchymal markers were present in freshly isolated SVF (Figures 1(a)–1(d)) and resulted to be highly enriched in ASCs derived from SVF *in vitro* culture for 15 days (Figures 1(e)–1(h)). As expected, the leukocyte population was present in SVF (Figure 1(d), CD45^+^ cells in the lower right side of the dot plot), but it was completely absent in the ASC culture after 15 days.

### 3.2. ASCs and SVF Differentiate into Osteoblasts *In Vitro*

**Figure 2:**
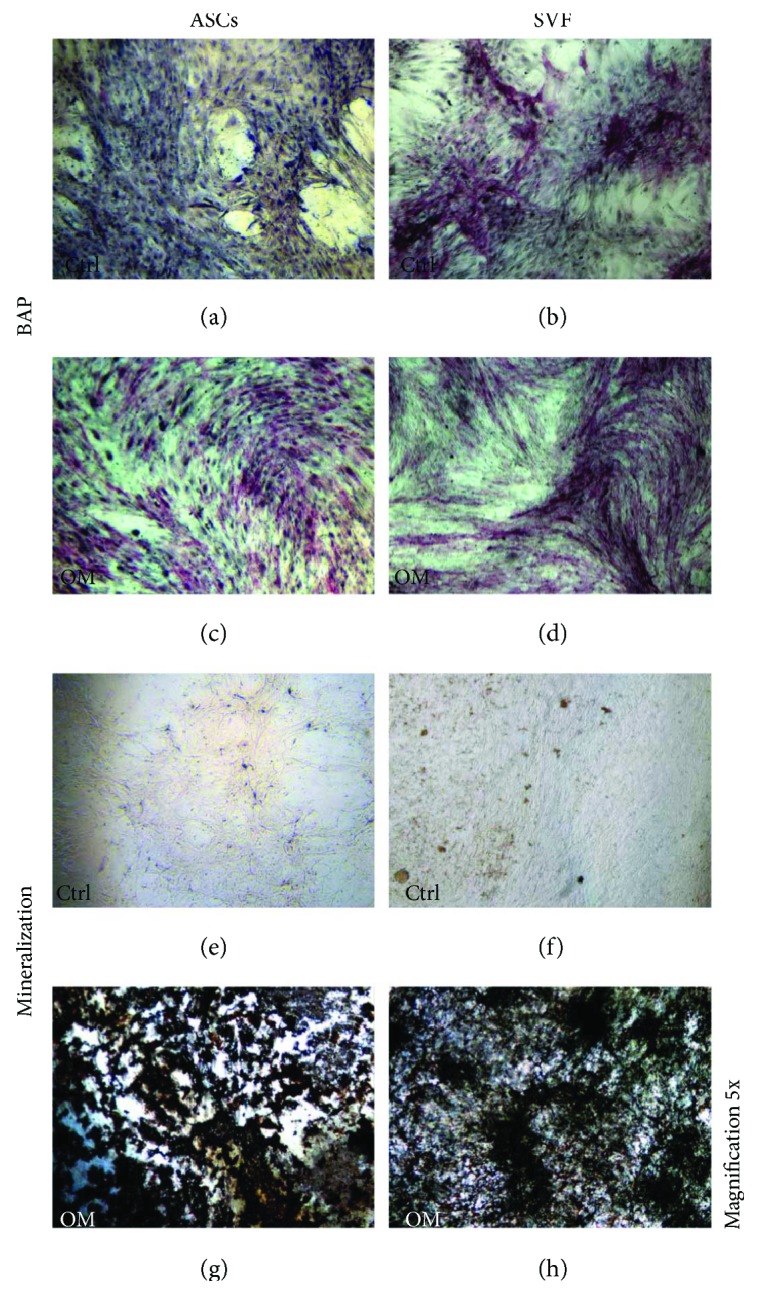
*In vitro* osteoblastic differentiation of ASCs and SVF. ASCs and SVF were cultured in the absence (ctrl) or in the presence of osteogenic medium (OM), for 60 days. In ctrl cultures, ALP expression is negative in ASCs (a), whereas it is positive in SVF (b). In the presence of OM, both ASCs and SVF show ALP-positive cells (c, d). For both ASCs and SVF, von Kossa staining is negative in the ctrl (e, f) and show mineral nodules with OM (g, h). Magnification 5x.

Both ASCs and SVF were cultured with or without OM for 60 days and showed differentiating ability towards osteoblasts (OBs) expressing ALP. Precisely, in the absence of osteogenic factors (control conditions), ASCs were ALP-negative (Figure 2(a)). On the contrary, ALP-positive cells were detected in SVF cultures, suggesting the presence of committed ASCs in SVF (Figure 2(b)). Both ASCs and SVF cultures with OM were ALP-positive (Figures 2(c)–2(d)). Next, we looked at the mineralization ability of both ASCs and SVF by von Kossa staining, as a readout of their activity. We observed that both types of cells did not mineralize in the absence of OM (Figures 2(e)–2(e)), whereas they did it in the presence of osteogenic factors (Figures 2(g)–2(h)).

### 3.3. ASCs and SVF Colonize SB

**Figure 3:**
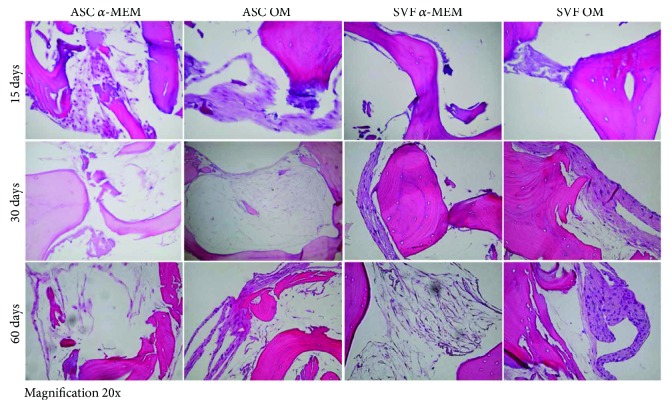
H&E staining to monitor SB colonization by ASCs and SVF. Both ASCs (left panels) and SVF (right panels) grow on SB in the absence of *α*-MEM or in osteogenic medium (OM). The presence of new tissue formation is evident since 15 days of culture, and it increases over time. Images of H&E staining are reported for each time point (15, 30, and 60 days). Magnification 20x.

H&E staining performed on SB cultured with ASCs and SVF showed the typical features of bone tissue, with trabeculae and empty lacunae, due to the complete decellularization of this biomaterial, particularly of its bovine-derived matrix, as described by the manufacturer [13]. Both ASCs and SVF colonized SB and formed new tissue on this biomaterial, starting from the periphery of the SB and filling bone lacunae, as described also in clinical studies [15]. We monitored the tissue growth at 15, 30, and 60 days, in both ASCs and SVF showing increasing areas of new tissue during the time, with and without OM, suggesting that SB is osteoinductive by itself (Figure 3).

**Figure 4:**
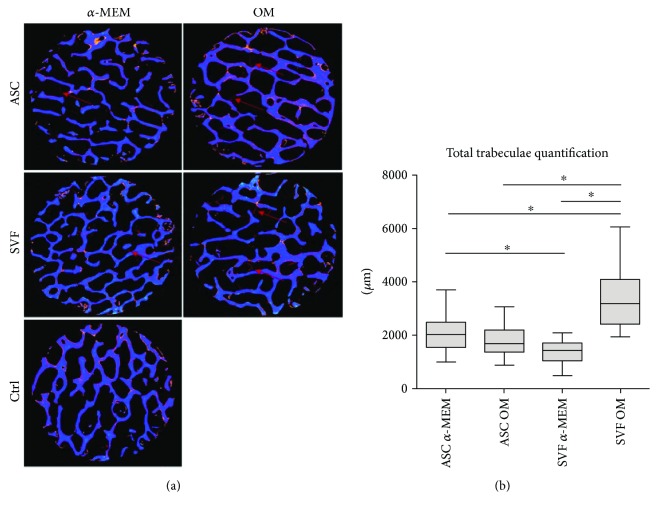
Analysis of new bone formation by micro-CT. (a) Representative images of X-ray tomography of SB with and without osteogenic medium (OM), after 60 days of culture with ASCs or SVF. The SB is shown in blue, whereas the newly formed mineralized tissue is in red (as indicated by the arrows). (b) Quantification of the newly formed mineralized tissue measured on SB cultured with ASCs or SVF, with or without OM. ^∗^ *p* < 0.05.

To monitor and quantify the growth of ASCs and SVF on SB, we assessed the presence of new tissue by micro-CT at different time points, showing a progressive increase in mineralized tissue apposition during time, from 15 to 60 days, both in the absence and in the presence of osteogenic factors. The newly formed bone-like segments present at 60 days are shown in Figure 4(a). The quantification analysis of the newly formed mineralized tissue demonstrated that SVFs were significantly more efficient than ASCs in inducing bone formation when cultured on SB with OM (Figure 4(a)). In the absence of osteogenic factors, SVF showed an osteogenic capacity significantly lower than ASCs cultured under both conditions. No significant differences could be detected between ASCs cultured on SB with or without OM (Figure 4(b)).

**Figure 5:**
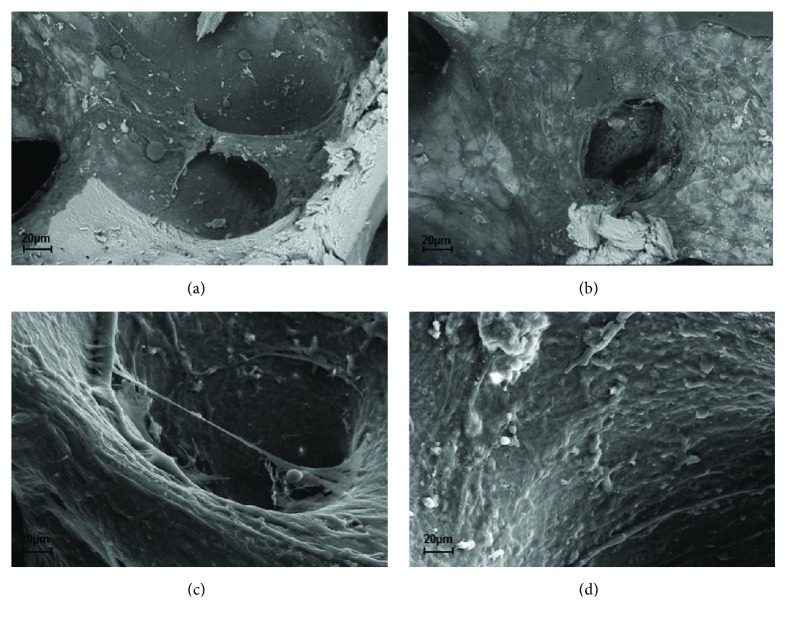
Analysis of new bone formation by SEM. Representative SEM images depicting SB after 30 days of culture with ASCs (a, c) and SVFs (b, d), respectively, at low (a, b) and high (c, d) magnifications.

SEM analyses also corroborated these data; indeed, we detected a marked new tissue formation on SB cultured with SVF (Figures 5(a) and 5(c)) compared to ASCs (Figures 5(b) and 5(d)) at 30 days and demonstrated a trend later detectable and quantified by micro-CT.

### 3.4. Expression of Osteoblast Markers by ASCs and SVF Cultured on SB

**Figure 6:**
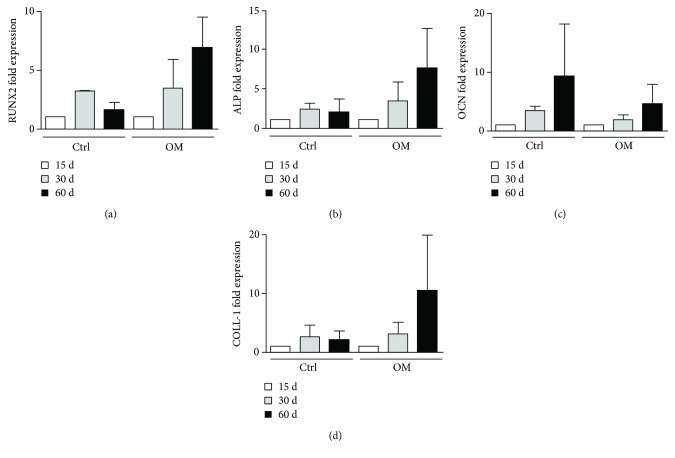
mRNA expression of osteogenic markers in ASCs and SVF plated on SB. The expression of RUNX2 (a), ALP (b), OCN (c), and COLL-1 (d) was analysed on ASCs and SVF plated on SB at 15, 30, and 60 days. A nonstatistically significant modulation of the expression of these genes was detected both without osteogenic factors (CTRL) and with OM.

To better characterise molecularly the cell growth on SB both in the absence (ctrl) and in the presence of OM, we evaluated the expression of an early osteogenic marker (Runx2) and of mature osteoblast markers (ALP, OCN, and COLL-1). We observed a modulation of the expression of all genes in ASCs and SVF cultures on SB at 30 and 60 days compared to our starting point (15 days). The highest increase in mRNA expression for all genes was observed at 60 days (Figure 6), even though no statistically significant differences were reported.

**Figure 7:**
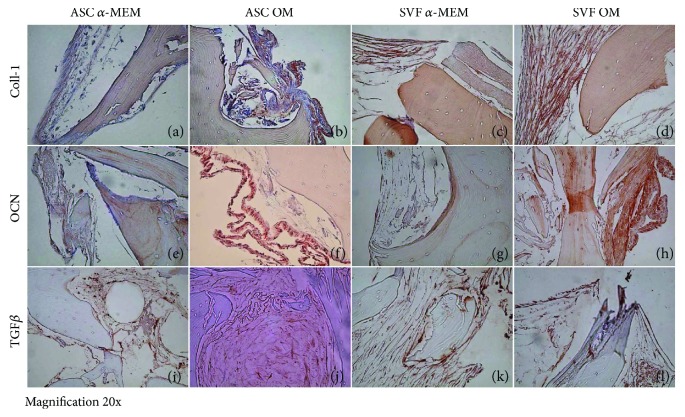
Osteoblast differentiation of ASCs and SVF on SmartBone. At 60 days, in ctrl condition, collagen I (COLL I) was weakly positive on SmartBone cultured with ASCs (a), whereas in culture with SVF, COLL I was expressed (c). Both in cultures with ASCs and SVF with osteogenic medium (OM), COLL I markedly stained the new tissue on SmartBone (b, d). Osteocalcin (OCN) was weakly positive in ctrl (e, g), and it was highly expressed with OM, mainly on the periphery of the newly formed tissue (f, h). TGFb stained osteoblasts at the margin of the newly formed tissue both with and without OM, with ASCs and SVF (i–l). Magnification: 20x.

To further confirm this result, we performed ALP, OCN, and TGF*β* staining on SB, which is negative for their expression by itself (Figure S1). We checked the expression of these proteins by cell growth on SB at 15 to 30 days of culture, observing a progressive increase in protein staining with the maximum expression at 60 days (Figure 7). Indeed, at 60 days, in control conditions, SB cultured with ASCs resulted to be weakly positive for COLL-1 fibers (Figures 7(a)), whereas in culture with SVF, COLL-1 was highly expressed (Figure 7(c)). In the presence of OM, both in cultures with ASCs and SVF, COLL-1 markedly stained the new tissue on SB (Figures 7(b) and 7(d)). OCN resulted to be weakly positive in controls (Figures 7(e) and 7(g)), whereas it was highly expressed with OM, mainly on the periphery of the newly formed tissue (Figures 7(f) and 7(h)). TGF*β* highlighted OBs at the boundaries of the newly formed tissue both with and without OM, with ASCs and SVF (Figures 7(i)–7(l)). These data confirm the potential of ASCs and SVF to differentiate into OBs, when cultured on SB.

### 3.5. Endothelin-1 and VEGF Are Produced by ASCs and SVF Plated on SB

**Figure 8:**
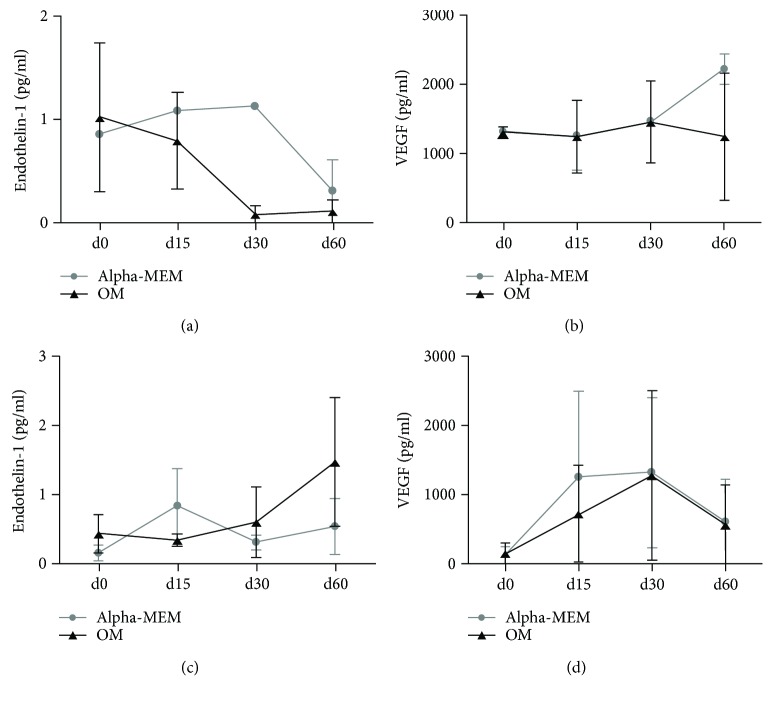
Secretion of ET1 and VEGF by ASCs and SVF plated on SB by ELISA assay. The levels of ET-1 and VEGF were dosed in cell culture supernatants at the beginning and during the culture. In ASC cultures, both ET-1 and VEGF were produced with and without osteogenic factors. ET-1 levels decreased, whereas high VEGF levels were constantly released over time (a, b). In SVF cultures, ET-1 showed an increasing trend of secretion with osteogenic factors, compared to a variable production in regular medium (c). VEGF levels increased until 30 days of culture, then they decreased with and without osteogenic factors (d).

The levels of ET-1 and VEGF were dosed in SB cell culture supernatants at the beginning and over the culture. In ASC cultures, both ET-1 and VEGF were produced with and without osteogenic factors. ET-1 levels tend to decrease at 60 days (Figure 8(a)), whereas high VEGF levels were constantly released over time (Figure 8(b)). In SVF cultures, the ET-1 level increased with osteogenic factors, whereas its production was variable with regular medium (Figure 8(c)). VEGF levels were increased until 30 days of culture, then they decreased both with and without osteogenic factors (Figure 8(d)). In both ASC and SVF cultures, with or without osteogenic factors, the levels of VEGF and ET-1 were not significantly different by statistical analysis.

## 4. Discussion

Human ASCs hold great potential for regenerative medicine applications. In this study, we demonstrated the efficacy of ASC-driven reconstruction of bone using SB, a xenohybrid bone graft scaffold. Importantly for clinical implications, we showed that SVF (the noncultured fraction of ASCs) has an osteoinductive ability on SB better than ASCs in the presence of osteogenic factors.

The regenerative ability of SVF derived from adipose tissue depends on soluble factors released and also on the presence in SVF of ASCs, which have multipotent differentiating capabilities. Indeed, SVF is a heterogeneous cell population containing endothelial cells, pericytes, leukocytes, red blood cells, and mesenchymal stem cells. ASCs can be derived from an *in vitro* cell culture of SVF. According to literature data, the ASC percentage in SVF is extremely different among patients, due to the human variability [17]. After 15 days of culture, we obtained an enrichment of the mesenchymal population initially present in SVF. ASCs expressed the typical mesenchymal markers CD105, CD73, CD44, and CD271 and were CD45-negative, as previously reported by literature data [3, 18–20]. Specifically, in our patients, the mean percentage of mesenchymal stem cells in freshly isolated SVF was 7%. The ability of ASCs to differentiate into OBs, chondrocytes, and adipocytes according to the different stimuli received has been deeply investigated by us and other groups [3, 5]. Here, we studied the osteogenic differentiating ability of ASCs and SVF, by comparing the ability to grow both in plastic and on SB, in regular and osteogenic media. Both ASCs and SVF differentiated into OBs expressing ALP and were able to mineralize when cultured in OM. ASCs were ALP-negative in the absence of osteogenic factors, whereas in SVF cultures we detected ALP-positive cells, suggesting the presence of committed ASCs in SVF. Even though osteogenic ALP-positive OBs were present in SVF, they did not mineralize, suggesting that microenvironmental factors play a key role in promoting the complete activation of these cells.

In order to improve bone regeneration, different biomaterials and scaffolds have been generated and tested: recent trends point towards a composite approach for best mimicking the human bone structure [12]. In this framework, we tested SB to study its supporting properties on mesenchymal cells and to evaluate the capabilities of ASCs and SVF to colonize and generate new tissue on it. After plating ASCs and SVF on SB, we monitored the tissue growth at 15, 30, and 60 days, showing increasing areas of new tissue over time with and without OM, suggesting that SB is osteoinductive by itself. By H&E staining, we showed that cells spread inside the SB scaffold, with a massive cell proliferation. The growth of ASCs and SVF on SB starts from the periphery of the SB, and then cells fill bone lacunas. The ability of mesenchymal cells to colonize and grow on SB, creating new tissue, explains and confirms the previously described osteointegrative capability of SB [15]. New bone formation within the bone substitute specimens was analysed recurring to micro-CT and SEM analyses. Interestingly, SVF cultured on SB resulted to be more effective than purified ASCs in promoting mineralized tissue apposition *in vitro*, based on the bone-like segment quantification of the scaffolds. Although the experimental setup is different and thus not entirely comparable, these findings are not completely in accordance with the *in vivo* study by Cheung et al. [21] who reported a similar mineral density for ASCs and SVFs. It should be underscored, however, that the data here presented may at least support the use of SVF to enhance inorganic bone substitutes, as recently proposed by Prins et al. [22], where SVF supplementation on either *β*-tricalcium phosphate or biphasic calcium phosphate carriers proved to be clinically useful.

To characterise molecularly the cells grown on SB in both regular and osteogenic media, we detected the expression of early markers of osteogenic differentiation of Runx2 and ALP and of mature osteoblast markers OCN and COLL-1 [23]. As expected, osteogenic medium increased the expression of all genes over time in our patients compared to medium without osteogenic factors, although this increase was not statistically significant between the two conditions. An enlarged panel of patients is needed to overcome the limitations of human variability and to give a statistical power to the analysis.

When we looked at the protein expression, we observed by IHC that in SVF culture, new tissue was markedly stained for COLL-1, OCN, and TGF*β* even in the absence of osteogenic factor stimulation, whereas in ASC culture the staining was detectable only with osteogenic medium. This result further confirms the micro-CT data on the high ability of SVF to colonize and generate new tissue on SB.

All those data open the debate on how it is possible that a smaller number of ASCs, present in freshly isolated SVF, compared to the expanded ASCs, could generate more tissue. We believe that all the different cell populations present in SVF likely cooperate and stimulate mesenchymal cell activity better than the ASCs alone, confirming the fundamental interplay between stem cells and the microenvironment. Indeed, previous studies had shown that human ASCs closely interact with vascular endothelial cells and secrete cytokines and growth factors (ASCs' secretome) into the extracellular milieu, with effects on different organs/systems within the human body [24, 25]. Among them, VEGF [26] and ET-1 [27] are paracrine factors secreted by ASCs that could promote OB differentiation. VEGF is not only a critical mediator in physiological angiogenesis but also a vital factor in skeletal growth. VEGF plays a positive role in the regulation of osteoblasts [28]: it has been reported that VEGF is expressed in osteoblast-like cells in a differentiation-dependent manner [29]. Studies on animal models showed how a combination of VEGF released from scaffolds previously seeded with bone marrow-derived MSCs (BM-MSCs) to the sites of bone damage resulted in increased regeneration of the bone defects [30]. Similarly, ET1 is involved in the regulation of osteogenic differentiation [31, 32], and it has been shown to be an important upregulator of MSC osteogenic and chondrogenic capacities [33, 34].

In order to better investigate the molecular signals responsible for successful bone regeneration in our model, we dosed VEGF and ET-1 secreted in cell culture supernatants over time, in the absence or presence of osteogenic factors. According to literature data, we observed a secretion of both VEGF and ET1 in MSCs after osteogenic differentiation *in vitro*. In particular, VEGF showed a more solid trend of increased secretion, while ET1 was more fluctuating among patients, likely due to human variability. These results confirm the importance of the adipose tissue-derived stem cell secretome in osteogenesis.

In conclusion, our data demonstrate for the first time that SVF has better osteoinductive capabilities than ASCs when plated on SB in osteogenic medium. Indeed, one of the major limitations of therapy with ASCs derives from the necessity to expand them in an *in vitro* culture, with the consequent necessity to comply with the restrictive regulatory clearance of cell therapy protocols (Good Manufacturing Practice). The absence of manipulation of SVF in an *in vitro* culture could definitively represent a benefit for a larger use.

SVF could be particularly useful in different clinical conditions, characterised by loss of bone and cartilage. The most common scenario is a localised osteocondral lesion of a large weight-bearing joint in patients who suffered trauma, osteocondritis of the growing joint, osteonecrosis, or oncologic resections. Defects of bone alone are also present in bone tumor procedures as curettage of common benign lesions such as fibrous dysplasia, giant cell tumor of bone, aneurysmal bone cysts, or unicameral bone cysts, whose defect after surgical procedure must be filled. Large bone defects or nonunion fractures in long bone are often treated locally, by injecting bone marrow-derived MSCs or ASCs in the fracture site with good clinical outcomes. The detractors of this technique criticise the difficulty in localising the stem cells in the selected site. Therefore, regenerative medicine based on a solid scaffold such as the SB, already available as a certified medical device, functionalised with SVF, could improve the precision of stem cell implants and the quality of new bone formation.

## Supplementary Materials

Supplementary Materials Fig. S1: ALP, OCN, and TGF*β* staining on SB. SB maintained in culture medium only was negative for the expressions of ALP, OCN, and TGF*β*.
