# Supplementary material for: Adipose-Derived Stromal Vascular Fraction/Xenohybrid Bone Scaffold: An Alternative Source for Bone Regeneration
Source: Stem Cells Int. 2018 Apr 29;2018:4126379. doi: 10.1155/2018/4126379 (PMC5949175; doi:10.1155/2018/4126379)
Supplement: Supplementary Materials — Fig. S1: ALP, OCN, and TGFβ staining on SB. SB maintained in culture medium only was negative for the expressions of ALP, OCN, and TGFβ. [file 4126379.f1.pdf]

Fig S1

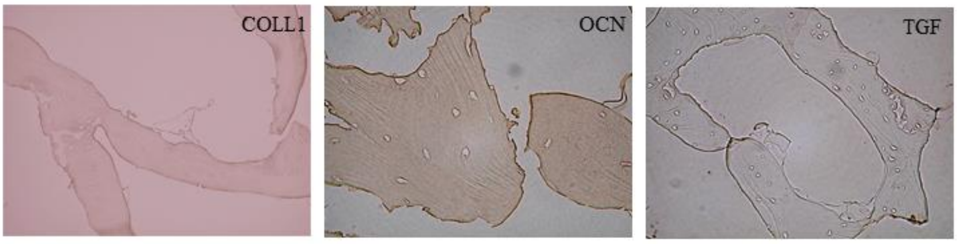

Magnification 20X

Fig S1. ALP, OCN and TGFβ staining on SB. SB maintained in culture medium only was negative for the expressions of ALP, OCN and TGFβ.
